# Interactions between horizontally acquired genes create a fitness cost in *Pseudomonas aeruginosa*

**DOI:** 10.1038/ncomms7845

**Published:** 2015-04-21

**Authors:** Alvaro San Millan, Macarena Toll-Riera, Qin Qi, R. Craig MacLean

**Affiliations:** 1 Department of Zoology, University of Oxford, South Parks Road OX1 3PS, Oxford, UK

## Abstract

Horizontal gene transfer (HGT) plays a key role in bacterial evolution, especially with respect to antibiotic resistance. Fitness costs associated with mobile genetic elements (MGEs) are thought to constrain HGT, but our understanding of these costs remains fragmentary, making it difficult to predict the success of HGT events. Here we use the interaction between *P. aeruginosa* and a costly plasmid (pNUK73) to investigate the molecular basis of the cost of HGT. Using RNA-Seq, we show that the acquisition of pNUK73 results in a profound alteration of the transcriptional profile of chromosomal genes. Mutations that inactivate two genes encoded on chromosomally integrated MGEs recover these fitness costs and transcriptional changes by decreasing the expression of the pNUK73 replication gene. Our study demonstrates that interactions between MGEs can compromise bacterial fitness via altered gene expression, and we argue that conflicts between mobile elements impose a general constraint on evolution by HGT.

## Abstract (web summary)

Horizontal gene transfer is important for bacterial evolution but the molecular basis of its fitness costs remain unclear. Here the authors show that fitness costs produced by a plasmid in *P. aeruginosa* are alleviated by mutations in recently acquired genes encoded in mobile genetic elements.

## Graphical abstract

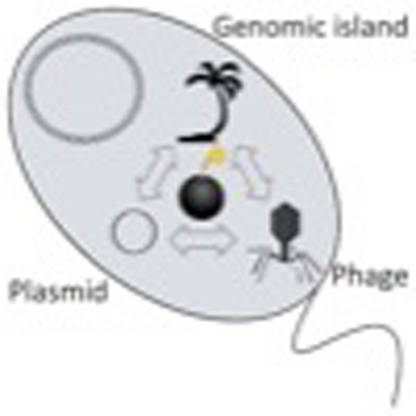

Horizontal gene transfer (HGT) is a key source of genetic diversity in bacteria, contributing to the adaptation and diversification of prokaryotes^1^,^2^. One recent and particularly concerning example of the ability of HGT to promote innovation in bacteria is the vast spread of antibiotic resistance among clinical pathogens^3^,^4^. However, the introduction of novel genes in a pre-existing, well-tuned genetic background is also a source of genetic conflict^5^. Recently acquired DNA can produce a fitness cost in the host bacteria^6^, but the origins and molecular mechanisms of these costs are poorly understood. The cost of HGT can arise from direct and indirect effects of the acquired DNA. For example, direct effects include the cost of the transfer process itself^7^, the disruption of the bacterial genome by the integration of foreign DNA^8^ and the metabolic costs associated with the replication, and more importantly the expression, of the newly acquired genes^9^,^10^. Indirect effects arise mainly from the interaction between the proteins encoded by the mobile genetic element (MGE) and the host^11^,^12^, which can lead to the disturbance of cellular networks^12^,^13^ and cytotoxic effects^14^. These interactions are especially relevant as shown by the fact that proteins with low levels of expression^15^ and low levels of connectivity with other proteins^16^,^17^ are more likely to be horizontally transferred. In addition, there are systems such as HN-S proteins, encoded both in the bacterial host^18^ and in MGEs^19^ that are able to silence recently acquired DNA and hinder these undesirable interactions. The costs of HGT will play a key role in the chances of establishment of newly acquired DNA in the host genome and in the bacterial population. Therefore, to understand the limits to evolution by HGT, it is crucial to understand the molecular basis of the cost of HGT.

In a recent study, we investigated compensatory adaptation between a small plasmid (pNUK73) and *Pseudomonas aeruginosa* PAO1^20^. The newly acquired plasmid produced a large fitness cost in the naïve bacterial host. However, after 300 generations of coexistence, PAO1 completely compensated for the cost of pNUK73 carriage. Whole-genome sequencing of several independent clones showed that mutations in any of three specific genes were responsible for the observed adaptation. The chromosomal genes that acquired compensatory mutations include a putative helicase carrying an UvrD-like helicase C-terminal domain (PA1372) and two contiguous putative serine/threonine protein kinases (PA4673.15 and PA4673.16; ref. 20). The modifications in these genes produced frameshifts and non-synonymous mutations, suggesting that the inactivation of any of these proteins was responsible for the compensatory adaptation to pNUK73.

In this study, we used the same model system to investigate the molecular basis of the fitness costs associated with HGT and how these costs can be alleviated by compensatory adaptation. Using RNA-Seq, we examine changes in the global transcriptional profile of the PAO1 host as a consequence of carrying the pNUK73 plasmid. We find that this plasmid produces a highly significant impact on the transcriptional profile of the host, including the induction of the SOS response via the expression of the plasmid replication protein gene *rep*. The previously identified compensatory mutations in the putative helicase and kinases reduce the expression of *rep* and restore the original transcriptional profile. Interestingly, we find that the putative helicase and kinases responsible for the increased expression of *rep* are recently acquired genes in *P. aeruginosa* carried on MGE and with no clear biological role in PAO1. Therefore, our results in the PAO1/pNUK73 system suggest that the interference between horizontally acquired elements drives the cost of HGT. We propose that this type of costly interactions between recently acquired MGE may be a general phenomenon in prokaryotes.

## Results

### Using RNA-Seq to dissect the cost of pNUK73

**Figure 1:**
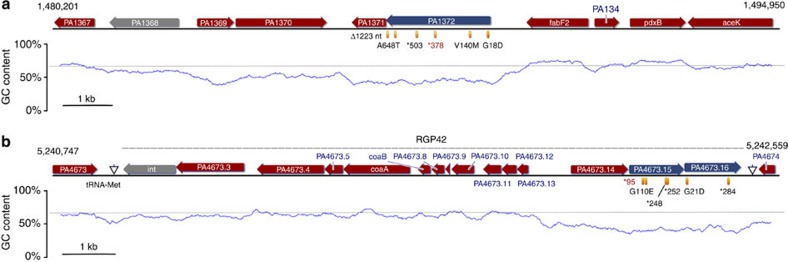
Genetic environment and mutations in PA1372 and PA4673.15-16. Loss-of-function mutations in three recently acquired genes compensate for the cost of pNUK73 in PAO1. The schematic diagrams show the genetic environment of (**a**) gene PA1372, a putative helicase and (**b**) genes PA4673.15-16 two contiguous putative serine/threonine protein kinases. Mutations in these genes are responsible for compensatory adaptation to plasmid pNUK73 in *P. aeruginosa* PAO1. The reading frames for genes are shown as arrows, with the direction of transcription indicated by the arrowhead. PA1372 and PA4673.15-16 are shown in blue. Genes involved in genetic transposition or integration are shown in grey. PA1368 is a transposase belonging to the IS4 family and PA4673.2 (*int*) is the RGP42 phage integrase. The rest of the genes are shown in red. The chart below the sequence represents GC content (blue line), with the black dotted line indicating the median GC content of the PAO1 genome (67%). The dashed line above the sequence in panel B indicates the position of the prophage RGP42 with the arrows pointing at the insertion site next to a tRNA-Met coding sequence. Yellow ellipses indicate the positions of the mutations responsible for compensatory adaptation to pNUK73 and the changes in the predicted protein sequence are described below them (an asterisk indicates a premature stop codon and Δ indicates a deletion). We found each one of these mutations independently in different clones that have compensated for the cost of plasmid pNUK73 from different populations. The clones used in this study are those with the mutations highlighted in red. Whole-genome sequencing showed that these two mutant clones carry no other modifications compared with parental PAO1/pNUK73 apart from the compensatory mutation. The numbers in the left and right above the sequences indicate the genomic location in the *P. aeruginosa* PAO1 genome (NCBI taxonomy ID: 208964).

**Figure 2:**
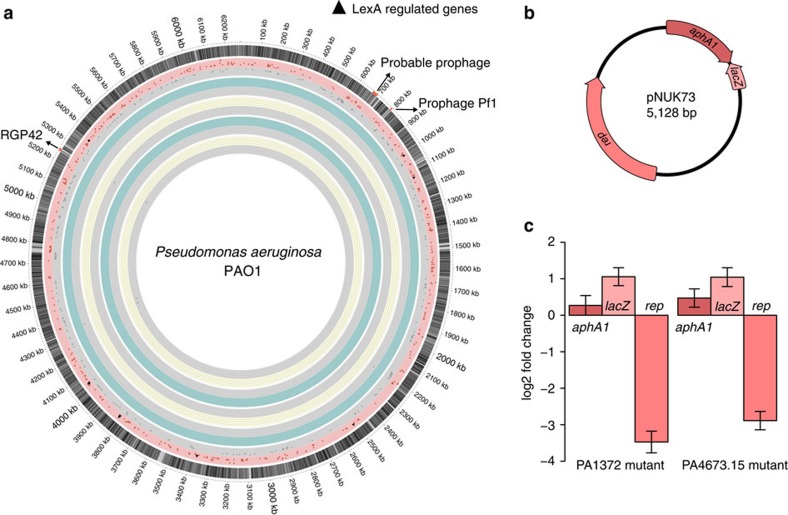
Gene expression map of the different strains analysed using RNA-Seq. The small plasmid pNUK73 produces a great impact on the transcriptional profile of PAO1 and compensatory mutations in the helicase or kinase decrease the expression of pNUK73 *rep* gene and restore PAO1 transcriptional profile. (**a**) Circos plot for the *P. aeruginosa* PAO1 genome. The outermost circle represents the GC content (light grey: GC content from 0 to 60%, grey: from 61 to 69%, dark grey: from 70 to 100%). From outside to inside, the 2nd to the 6th circles illustrate the changes in the transcriptional profiles in the following strains relative to wild-type PAO1. Within each circle, a scatterplot is drawn, with lines representing the fold-change (log_2_) relative to PAO1. Each dot in the scatterplot represents a differentially expressed gene for each particular comparison. Triangles represent LexA-regulated genes. Two biological replicates of RNA samples obtained on different days were sequenced for each strain. 2nd circle: parental PAO1/pNUK73 (red: up-, grey: downregulation). 3rd circle: helicase mutant (PA1372)/pNUK73 (green: up-, grey: downregulation). 4th circle: kinase mutant (PA4673.15)/pNUK73 (yellow: up-, grey: downregulation). 5th circle: plasmid-free helicase mutant (PA1372) (green: up-, grey: downregulation). 6th circle: plasmid-free kinase mutant (PA4673.15) (yellow: up-, grey: downregulation). (**b**) Schematic representation of the pNUK73 plasmid. The reading frames for genes are shown as arrows, with the direction of transcription indicated by the arrowhead. (**c**) Differences in expression of pNUK73 genes in the helicase and kinase mutant strains compared to PAO1/pNUK73 (log_2_ scale, *N*=3). The error bars represent the standard error of the mean.

As a first approach in understanding the molecular basis of the cost produced by pNUK73 in PAO1, we used RNA-Seq to analyse the genome-wide transcriptional profiles of PAO1 and PAO1 carrying PNUK73 plasmid (PAO1/pNUK73). In addition, to understand the contribution of transcriptional changes to compensatory evolution, we performed RNA-Seq on two pNUK73-bearing PAO1 mutants carrying two different compensatory mutations in the genome. One compensatory mutation was found in a putative helicase gene (PA1372 with premature stop codon at position 378, Fig. 1), while the other was in a putative kinase gene (PA4673.15 with premature stop codon at position 95, Fig. 1). The mutant clones carried no other mutations compared with the parental PAO1/pNUK73 apart from the compensatory mutation^20^. We found that pNUK73 acquisition significantly altered the expression of 749 genes in the host genome (Wald Test Benjamini-Hochberg corrected *P*-value<0.05, *n*=5,715), which represent 13% of the total number of genes in PAO1 (Fig. 2a, Supplementary Data 1). Gene set enrichment analyses (see Methods) showed that upregulated genes are mainly enriched in the functional classes of translation, ribosomal structure and biogenesis, while downregulated genes are enriched in metabolism (Supplementary Data 2).

Interestingly, we found that plasmid-bearing strains carrying compensatory mutations in either the helicase or the kinase genes showed transcriptional profiles that were almost identical to those of plasmid-free PAO1 with a few very minor exceptions (Fig. 2a). In the helicase mutant, only two genes were significantly downregulated, PA2026 and PA3877, which encode a putative Na^+^-dependent transporter and a nitrite extrusion protein, respectively (Supplementary Data 1). In the plasmid-bearing kinase mutant clone one single gene was significantly downregulated: PA4673.16, which codes for the second contiguous putative kinase that can also be mutated to compensate for the cost of pNUK73 carriage (Fig. 1). PA4673.15 itself was also underexpressed, albeit non-significantly, suggesting that PA4673.15 regulates the expression of both kinases.

The pNUK73 small plasmid carries the backbone of the natural cryptic plasmid pNI10 isolated from *Pseudomonas fulva*^21^ and codes for three genes, namely the Rep plasmid replication protein gene from pNI10 (*rep*), an aminoglycoside 3′-phosphotransferase gene (*aphA1*, which confers resistance to kanamycin and neomycin) and a *lacZ* reporter gene (Fig. 2b). We compared the expression levels of these three genes between the parental PAO1/pNUK73 and the plasmid-bearing compensated mutants (Fig. 2c). We observed a marked reduction on *rep* expression, which decreased 11.1-fold (Wald Test Benjamini–Hochberg corrected *P*<10^−28^, *n*=5,715) and 7.4-fold (Wald Test Benjamini–Hochberg corrected *P*<10^−27^, *n*=5,715) in the helicase and kinase mutant, respectively, relative to parental PAO1/pNUK73 (Fig. 2c). The expression levels of *aphA1* remained unchanged in both compensated mutants, while *lacZ* was slightly overexpressed (ca. 2.1-fold in both clones: Wald Test Benjamini–Hochberg corrected, helicase mutant *P*=0.0002; kinase mutant *P*=0.0004, *n*=5,715). Despite the significant decrease in the expression of *rep*, we showed in our previous work that the copy numbers of pNUK73 in clones with helicase (*n*=2) and kinases (*n*=4) mutations (average=7.77, s.e.=0.79) were not significant different to that in the PAO1/pNUK73 parental strain (average=11.03, s.e.=1.89, Two-sample *t*-test: *P*=0.171, *t*=1.59, df=5.21; ref. 20). These results suggest that the expression level of *rep* in parental PAO1/pNUK73 may be unnecessary high compared with those in the compensated mutants.

The correlation between decreased *rep* expression levels and restored expression of chromosomal genes observed in the compensated mutants suggests that *rep* expression may be linked to both the cost of plasmid carriage and the genome-wide impact of pNUK73 on the expression of chromosomal genes (Fig. 2). In an elegant study, Ingmer *et al*.^22^ showed how the plasmid replication protein RepA encoded by the small plasmid pSC101 induces a delay in *E. coli* host cell division by activating the SOS response^22^. RepA binds primarily to its binding sites in pSC101 and recruits host replication proteins such as DnaA, DnaB and DnaG. However, when there is an excess of free RepA in the cell it sequesters DnaG, stalling chromosomal replication and inhibiting cell division by inducing the SOS response. It is thought that this mechanism may help to limit the rate of segregational loss of the pSC101 plasmid by delaying cell division in host cells carrying low copy numbers of this plasmid (and therefore low number of RepA binding sites and high levels of free RepA)^22^.

Consistent with the model proposed by Ingmer and colleagues, we found that pNUK73 induces the expression of the SOS response. Ten out of the 15 known LexA-regulated genes in *P. aeruginosa*, including *recA* and *lexA*, were significantly overexpressed in PAO1/pNUK73 compared to PAO1, and the remaining five also showed a non-significant increase in their expression levels (Fig. 2a, Supplementary Table 1)^23^. Furthermore, plasmid carriage engendered a significant reduction in fitness consistent with a delay in host cell division^20^. Finally, as explained above, we found that compensatory mutations in the helicase and kinase both reduced *rep* expression and silenced the expression of the SOS response.

### Linking Rep expression to the cost of plasmid carriage

**Figure 3:**
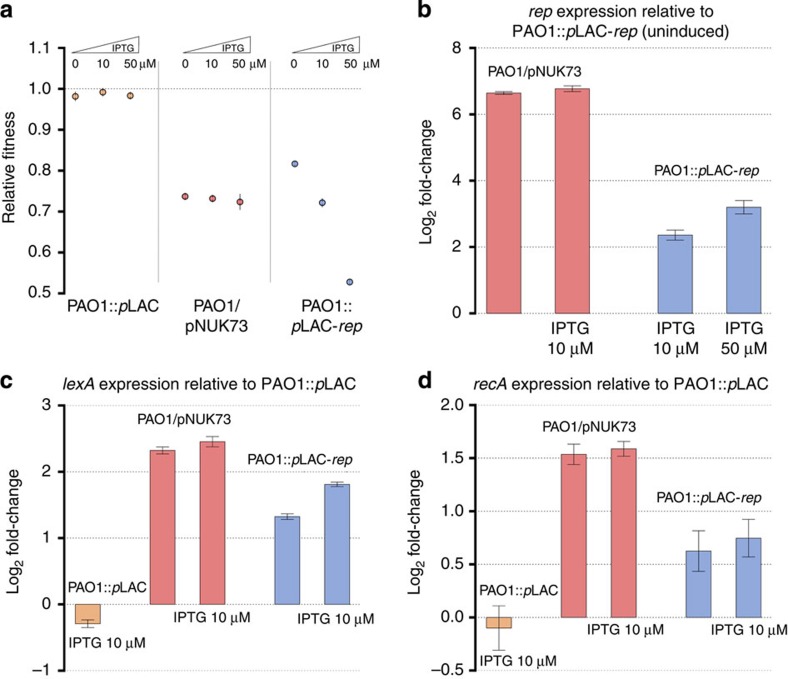
Expression of *rep* reduces fitness and activates the SOS response in PAO1. This figure shows how the plasmid replication protein is responsible for fitness cost and activation of SOS response in PAO1. (**a**) The relative fitness compared to plasmid-free wild-type PAO1 for the PAO1::*p*LAC negative control strain (orange), parental PAO1/pNUK73 (red), and PAO1::*p*LAC-*rep* (blue) under IPTG concentrations of 0 μM (first point), 10 μM (second point) and 50 μM (third point). The error bars represent the s.e.m. (*N*=3). (**b**) The relative expression (log_2_ fold change, ±s.e.m., *N*=3) of the *rep* gene relative to the uninduced PAO1::*p*LAC-*rep* strain in PAO1/pNUK73 (0 and 10 μM IPTG) and PAO1::*p*LAC-*rep* (10 and 50 μM IPTG). The expression levels (log_2_ fold-change, ±s.e.m., *N*=3) of *lexA* (**c**) and *recA* (**d**) relative to the PAO1::*p*LAC negative control strain. Expression measurements were carried out in the presence of 10 μM IPTG and without IPTG induction. Each measurement was performed independently at least three times and the error bars represent the s.e.m.

To test the hypothesis that *rep* expression is directly responsible for the fitness cost associated with pNUK73 carriage, we cloned the *rep* gene under the control of an isopropyl β-D-1-thiogalactopyranoside (IPTG)-inducible *lac* operon promoter (*p*LAC) and integrated this inducible *rep* construct into the PAO1 genome using the mini-Tn7 system^24^ to generate the PAO1::*p*LAC-*rep* strain. Using the same system, we cloned *p*LAC into the PAO1 genome to generate the PAO1::*p*LAC negative control strain. To determine the effect of *rep* expression on fitness, we directly competed these two strains as well as the parental PAO1/pNUK73 against a plasmid-free, GFP-tagged PAO1 strain across different IPTG concentrations (Fig. 3a). The negative control strain did not show any reduction in fitness relative to the parental PAO1 under any induction regime, confirming that insertion of *p*LAC at the mini-Tn7 chromosomal site has no deleterious effects on the fitness of PAO1 (analysis of variance: *P*=0.243, F=1.427, df=1, 27). On the other hand, PAO1::*p*LAC-*rep* showed a significant reduction in relative fitness, even in the absence of IPTG induction (average fitness=0.817, s.e.=0.005, *n*=3, two-sample *t*-test: *P*<0.001, *t*=16.2, df=7.8). The cost of *rep* expression increased with IPTG concentration, and at the highest IPTG concentration (50 μM), the cost of *rep* construct expression (average fitness=0.528, s.e.=0.004, *n*=3) was even higher than that of pNUK73 carriage in the ancestral PAO1 strain (average fitness=0.737, s.e.=0.008, *n*=4, two-sample *t*-test: *P*<0.001, *t*=22.2, df=4.3).

To link the fitness cost associated with *rep* expression to altered gene expression, we quantified the expression levels of *rep* and the SOS response genes *lexA* and *recA* in PAO1::*p*LAC-*rep* and PAO1/pNUK73 by quantiatative PCR(qPCR). We found that the absolute transcript levels of *rep* stemming from leaky expression in the absence of IPTG was 7.35% (±0.86) that of *rpoD* (*n*=3), a constitutively expressed essential gene whose expression is estimated to give rise to 1,500 molecules of the RpoD protein per cell^25^,^26^. This relatively high level of constitutive, leaky expression helps to explain why the fitness of the PAO1::*p*LAC-*rep* showed a reduction relative to the negative control strain even in the absence of IPTG induction. As expected from the fitness data, adding IPTG led to significant increases in *rep* expression (0 versus 10 μM IPTG, two-sample *t*-test: *P*=0.01, *t*=8.1, df=2.21, Fig. 3b). The expression of SOS regulated genes in PAO1::*p*LAC-*rep* strain essentially mirrored the expression of *rep*. Constitutive leaky expression of *rep* induced the expression of *lexA* and *recA*, and IPTG induction resulted in a modest increase in the expression of SOS regulated genes (Fig. 3c,d). Consistent with our RNA-Seq data, *rep, lexA* and *recA* were expressed at high levels in PAO1/pNUK73 (Fig. 3b–d). These qPCR data broadly support our phenotypic measurements of competitive fitness, and confirm that increased *rep* expression is associated with decreased fitness and changes in *lexA/recA* expression that parallel those associated with pNUK73 carriage. The causality of this relationship was established by our RNA-Seq data, which revealed that compensatory mutations reduced the expression of *rep*. In addition, the upregulation of the SOS response genes shown by the gene expression analyses by qPCR and RNA-Seq support the idea that the SOS regulon is activated concomitantly with *rep* expression. In agreement with the gene expression analyses, we found that the presence of pNUK73 or the chromosomal copy of *rep* produced an increase in PAO1 cell size, which is associated with the delay in cell division induced by the SOS response^27^ (Supplementary Fig. 1).

At the same time, it is also evident that there are some quantitative differences between the PAO1/pNUK73 and PAO1::*p*LAC-*rep* strains with respect to how variations in *rep* expression levels can translate into differences in fitness costs. For example, *rep* expression in the presence of 10 μM IPTG in PAO1::*p*LAC-*rep* resulted in a fitness cost that is comparable to that of pNUK73 carriage (two-sample *t*-test: *P*=0.317, *t*=1.13, df=4.2). However, the relative expression levels of *rep* were higher in PAO1/pNUK73 than in the IPTG-inducible strain (Fig. 3). We can infer that pNUK73 neutralizes some of the deleterious effects associated with *rep* expression, probably by sequestering Rep protein on plasmid-carried Rep binding sites, which has been shown by Ingmer *et al*.^22^.

### The helicase and kinases are recently acquired genes in PAO1

Bioinformatic analyses show that the helicase (PA1372) and kinases genes (PA4673.15-16) that have acquired compensatory mutations exhibit a strong signature of recent horizontal acquisition in PAO1. First, while the guanine+cytosine (GC) content of the *P. aeruginosa* PAO1 genome is very high (median content of PAO1 genes=67%), these three genes have particularly low GC contents (44% in PA1372; 40% in PA4673.15; 42% in PA4673.16, Fig. 1), and fall in the bottom 1 percentile of GC content in the PAO1 genome (Supplementary Fig. 2). Second, the three genes seem to be located in potentially mobile DNA regions. PA4673.15-16 genes are located inside the prophage RGP42^28^, and PA1372 is found inside a small low GC content island close to a gene coding for a putative transposase (PA1368) (Fig. 1). Finally, these genes are very rare in all sequenced genomes of *P. aeruginosa*. Out of the 18 *P. aeruginosa* complete annotated genomes available in GenBank, PA1372 is found only in 3, while PA4673.15-16 are absent. These results indicate that the compensatory mutations responsible for the adaptation to pNUK73 occurred in genes that have been recently acquired by *P. aeruginosa* PAO1. Moreover, when performing similarity searches of these proteins against the non-redundant database of NCBI and against the European Nucleotide Archive, the hits were found to have very low percentage of identity (50% or lower) in other species. Interestingly, in those species where a hit was found, the genetic context was the same as in *P. aeruginosa* PAO1, that is, PA4673.15-16 conserved their synteny and were preceded by PA4673.14, while PA1372 was found together with PA1371. In addition, mobile DNA genes surrounded PA4673.14-16 and PA1731-2 in those genomes (that is, recombinase, phage integrase and transposase). These results suggest that both PA1371-2 and PA4673.15-16 are genomic regions of unknown origin and very prone to be horizontally transferred.

Mobile genetic elements can spread through bacterial populations by acting as symbionts that increase the fitness of their bacterial hosts or by acting as parasites that selfishly exploit their hosts to maximize their own transmission^29^. Of course these two strategies are not mutually exclusive and many mobile elements can probably act as both symbionts and parasites, but it is important to draw this distinction because the evolutionary interpretation of the conflict between pNUK73 and the helicase or kinases depends crucially on whether these elements are symbionts or parasites. Previously, we have shown that mutations in the helicase and kinase do not alter bacterial fitness under laboratory conditions^20^, suggesting that these genes are parasites, and not symbionts. To further test this idea, we measured the impact of helicase and kinase mutations on gene expression in the absence of pNUK73 carriage using RNA-Seq (Fig. 2a). We found that the plasmid-free compensated mutants had transcriptional profiles that were almost identical to wild-type PAO1. We did not detect any differentially expressed genes in the plasmid-free helicase mutant, and only four genes were differentially expressed in the kinase mutant, including the two putative kinases (PA4673.15-16), a molybdenum cofactor biosynthetic protein A1 (PA3194) and a nitrite extrusion protein (PA3887). These last two genes were underexpressed in both the plasmid-bearing helicase mutant and the plasmid-free kinase mutant (Wald Test Benjamini–Hochberg corrected, PA3887 *P*=0.044 and 0.008, respectively; PA3194 *P*=0.073 and 0.005, respectively, *n*=5715, Supplementary Data 1), suggesting that the kinase and the helicase may interact with each other.

Collectively, these transcriptomic results support the idea that these helicase and kinase genes do not play any functional role in *P. aeruginosa*, at least under laboratory conditions, perhaps because they are recently acquired genes that are still not integrated in the transcriptional regulatory network of the bacterium. To further investigate this idea at a phenotypic level, we carried out two experiments designed to test for a functional role of the helicase and kinase genes. First, we tested the impact of helicase and kinase mutations on bacterial growth across a wider range of biological conditions using Biolog EcoPlates. We failed to find any disadvantage in average growth rate or stationary phase density associated with the helicase or kinase inactivating mutations in any of the tested environments (Supplementary Fig. 3). In fact, both plasmid-free mutants showed a slight increase in growth rate compared with the wild-type strain, which supports the idea of their absence of biological role in PAO1. Second, to test if these mutations played a general role in adaptation to plasmids in PAO1, we analysed the impact of helicase and kinase mutations on the fitness burden associated with carrying four plasmids: Rms149^30^, pAKD1^31^, PAMBL-1 and PAMBL-2^32^. We found no difference in the fitness burden associated with carrying these plasmids in the helicase and kinase mutants compared with the wild-type strain, indicating that these mutations are not general adaptations to plasmids (Supplementary Fig. 4).

## Discussion

In this study, we used *P. aeruginosa* PAO1 and the small plasmid pNUK73 as a model system to investigate the origin of the costs of HGT and the nature of its subsequent compensatory adaptation. We found that the cost of pNUK73 was generated by the expression of the plasmid replication protein gene *rep*, which in turn produced massive changes in the expression of the PAO1 genome (749 genes), including the activation of the SOS response and the expression of genes that are associated with stalled chromosomal replication. Remarkably, the alteration produced by pNUK73 in the expression of PAO1 genome is larger than the effect of general transcriptional regulators such as LasR or AmpR^33^,^34^. The high-level expression of *rep* depends on the presence of a putative helicase (PA1372) and two putative protein kinases (PA4673.15-16), which showed strong signatures of HGT and no apparent biological role in PAO1. The inactivation of the helicase or one of the kinases genes completely restored the changes in gene expression and fitness associated with pNUK73 acquisition. To the best of our knowledge, this is one of the few studies dissecting the mechanistic basis of cost and adaptation in a bacterium/mobile element interaction at a genetic and transcriptomic level^35^.

One of the most important limitations of this study is that although we clearly showed the link between the mutations in the helicase and kinase and the reduction of *rep* expression, we could not elucidate the specific interactions among these genes driving the high level expression of *rep*. Our results suggest that the helicase and kinase interact with each other, since mutations in both helicase and kinase lead to the underexpression of PA3194 and PA3887 genes. In addition, helicases are known to interact with plasmid replication proteins^36^,^37^. Taken together, these lines of evidence suggest that the kinases may be responsible for the activation of the helicase through phosphorylation, which could in turn interact with pNUK73 replication protein, leading to the derepression or activation of *rep* expression. Further experimental work will be necessary to elucidate the nature of these interactions.

Our results clearly show that the interactions between recently acquired genes are responsible for the cost of HGT in this model. Interactions between MGEs have previously been shown to influence bacterial fitness; for example, epistatic interactions between co-occurring plasmids in the same bacterial strain have been shown to both buffer and aggravate the fitness cost of plasmid carriage^38^,^39^. Unfortunately, the mechanistic basis of epistasis has not been elucidated in these studies. Interactions between MGEs that influence fitness should play a key role in the persistence of MGEs in bacterial populations; positive interactions that ameliorate the cost of MGE carriage should increase the stability of MGEs in bacterial populations, while negative interactions that exacerbate the costs of MGE carriage should drive the loss of mobile elements.

Why do mobile elements interact with each other? One possibility is that MGEs have specifically evolved systems to cooperate or compete with other MGEs (for examples, see refs 40, 41), and the interactions between them could translate into a fitness alteration for the host. This explanation requires an evolutionary history between the MGEs. Alternatively, it is possible that interactions between MGEs arise as a spurious accident. By definition, HGT brings together genes that have different evolutionary histories, and there is no *a priori* reason to expect that these genes should interact with each other in a mutually beneficial way. Even if the different acquired genes have evolved together and could interact with each other, it is likely that these interactions would produce different results in a new species compared to in the original host due to the different genetic circuitry. Therefore, under the accident hypothesis the recently acquired genes are likely to produce negative effects on the host. We argue that our model system provides an example for the accident hypothesis. We found that although the helicase and kinases seemed to interact with each other, they produced a negligible impact on the expression profile and fitness of PAO1, essentially acting as pure genetic parasites. In the presence of pNUK73, however, these two genes interact to induce high-level expression of the pNUK73 Rep protein, triggering cytotoxic effects that lead to tragic consequences for all the parties involved in this interaction. In fact, it is only in combination that the three acquired genes produced a big cost in PAO1, while any pairwise combinations produced no extra cost^20^.

We speculate that these types of costly accidental interactions between MGE are probably frequent in bacteria and may restrict evolution via HGT. The precise molecular bases of these interactions are difficult to predict due to the lack of experimental evidence in current literature. However, it is likely that the fitness cost derived from these accidental interactions is generally based on the destabilization of fine-tuned host cellular networks such as DNA replication, as is the case in our model system.

In this work, we have elucidated the mechanisms implicated in the cost of the small plasmid pNUK73 in *P. aeruginosa* PAO1, as well as the mechanistic basis for the alleviation of this cost via compensatory evolution. We found that the detrimental effects produced by this plasmid arose from interactions between recently acquired genetic elements in PAO1. Even though the results presented here are only applicable to this model system, several lines of evidence support the idea that the interactions between recently acquired genetic elements may play a central role in the cost of HGT. Future experimental and bioinformatic work will be necessary to provide further support to this hypothesis.

## Methods

### Bacterial strains and plasmids and culture conditions

All bacterial strains were cultured in LB broth at 37 °C with continuous shaking (225 r.p.m.) and on LB agar plates at 37 °C (Fisher Scientific, USA). Plasmids were electroporated into PAO1 and plasmid-bearing colonies were selected as previously described^39^. The small plasmid pNUK73 does not belong to any defined incompatibility group^21^. Rms149 belongs to the incompatibility group IncP-6, while pAKD1 belongs to the IncP-1β group^30^,^31^. PAMBL-1 and PAMBL-2 complete sequences are not available and their incompatibility groups are not determined using the PCR replicon typing system^42^. We used Biolog EcoPlates (Biolog, USA) to assess bacterial growth in different environments according to the manufacturer’s instructions using a BioTek Synergy H4 plate reader (BioTek Instruments, UK). Competitive fitness experiments were performed and analysed as described in San Millan *et al*.^20^: Briefly, we precultured the strains at 37 °C with 225 r.p.m. shaking overnight in 3 ml of LB broth (Fisher Scientific, USA). We diluted 10 μl of the pre-cultures in 190 μl of fresh LB and incubated in the same conditions in 96-well plates until they reach mid-exponential phase (OD_600_ of≈0.5). We mixed these cultures at a ratio of approximately 50% clone under study to 50% PAO1–GFP. We confirmed the initial proportions using flow cytometry with an Accuri C6 Flow Cytometer Instrument (BD Accuri, USA). We diluted the mixtures 400-fold in fresh LB and cultured for 16 h at 37 °C with 225 r.p.m. shaking (∼8 generations). We measured the final proportion using flow cytometry again. Flow cytometry was performed using an Accuri C6 Instrument (BD Accuri, USA) with the following parameters: flow rate: 66 μl min^−1^, core size: 22 μm, events recorded per sample: 10,000–20,000.

### RNA extractions and reverse transcription

Each bacterial strain (Supplementary Table 2) was inoculated in LB medium with IPTG induction (Sigma-Aldrich, USA) where required and grown overnight at 37 °C with continuous shaking (225 r.p.m.). The overnight cultures were diluted 1:100 in fresh LB with the same concentration of IPTG and incubated until they reached an OD_600_ of 0.5 under the same experimental conditions. Bacterial cultures were mixed with RNAprotect Bacteria Reagent (Qiagen, Netherlands) according to the manufacturer’s instructions. Total RNA extraction was performed using the SV Total RNA Isolation System (Promega, USA). To eliminate genomic DNA, an on-column DNase I digest (Promega, USA) and an additional DNase treatment using the Ambion Turbo DNA-free kit (Life Technologies, USA) were performed during and after the RNA isolation procedure, respectively, according to the manufacturers’ instructions. For reverse transcription, first-strand cDNA samples were synthesized from 2.0 μg of total RNA templates using the GoScript Reverse Transcription System with random hexamer primers (Promega, USA). Negative control reactions, which contained all components for reverse transcription with the exception of the reverse transcriptase enzyme, were performed to verify the absence of genomic DNA in the RNA samples.

### RNA-Seq analysis

RNA-Seq analysis was performed on the transcriptomic data of six strains: PAO1 wild-type, parental PAO1/pNUK73, and two compensated mutants both carrying the plasmid and cured: a helicase mutant clone (PA1372, premature stop codon at position 378) and a kinase mutant clone (PA4673.15, premature stop codon at position 95). The mutant strains carried no other mutations in the genome compared with the parental PAO1 apart from the ones specified above, as shown by whole-genome sequencing^20^. Two biological replicates of RNA samples obtained on different days were sequenced for each strain as described above. Library preparation (directional paired-end ribodepleted library) and sequencing (using Illumina MiSeq) were performed at the Wellcome Trust Centre for Human Genetics, University of Oxford. Library quality was assessed using the 2200 TapeStation Software (Agilent Technologies, USA) and the Qubit Fluorometric Quantitation platform (Life Technologies, USA). Raw reads were initially filtered using the NGS QC Toolkit^43^, discarding on average 7.8% of the reads per sample. The 3′ and 5′ ends of the reads were trimmed if the Phred quality score was <20, and any reads shorter than 75 bp were discarded after the trimming step. Subsequently, only reads with a Phred quality score of at least 20 throughout 80% of their lengths were retained. Finally, reads that presented ambiguous values in >2% of their bases were discarded.

The PAO1 ancestral strain used in this study has two additional features compared with the *P. aeruginosa* PAO1 reference genome (NC_002516.2), namely the insertion of the phage RGP42 (GQ141978.1) and the plasmid pNUK73 (AB084167). Filtered reads were mapped to the *P. aeruginosa* PAO1 reference genome and to the reference sequences for the pNUK73 plasmid and the RGP42 phage using BWA^44^. The average coverage was 43.5 × , and 98.2% of the bases had a Phred quality score of 20 or higher. Gene counts were obtained from BAM files using *HTSeq*^45^ (options: —m (mode to handle overlapping reads), intersection-strict, —a (minimum read quality alignment), 20). Differential gene expression analysis with correction for the batch effect was performed using *DESeq2*^46^ (function DESeqDataSetFromHTSeqCount). The design formula design=∼batch+condition was used to test for the effects of each condition (helicase mutated, kinase mutated, plasmid bearing and ancestral strain), controlling for putative sources of variation due to sample preparation on different days (the batch effect). The FASTQ files generated in this work have been deposited in the European Nucleotide Archive database under the accession code PRJEB8227.

### Quantitative real-time PCR

The experimental procedures for quantitative real-time PCR (rt-qPCR) were modified from those described in a study by Qi *et al*.^47^. The rt-qPCR assays were performed using the relative quantification method. Each gene shown in Supplementary Table 3 was amplified using the Fast SYBR Green Master Mix (Applied Biosystems, USA) and 100 nM oligonucleotide primers on the StepOnePlus Real-time PCR platform (Applied Biosystems, USA) in three biological replicates and two technical replicates. The amplification efficiency, linearity and specificity of each qPCR primer pair were verified three times using a serially diluted pool of experimental cDNA samples (Supplementary Table 3). The transcript levels (expressed in log_2_ fold-change) of the target genes *lexA*, *recA* and *rep* in all the test strains were determined relative to the relevant control strains, that is, the PAO1::*p*LAC control strain for *lexA* and *recA* expression; the PAO1::pLAC-*rep* strain without IPTG induction for *rep* expression. Normalization factors were calculated from the geometric mean of the transcript levels of three stably expressed internal reference genes (*acpP*, *atpA* and *rpoD*). The transcript levels of the target genes were determined by normalizing the transcript levels of every target genes to the normalization factors. Finally, the relative transcript levels for each target gene in each sample group were calculated after correcting for the batch effect.

To quantify the extent of leaky expression of *rep* from *p*LAC, we measured the copy number of *rep* and *rpoD* transcripts in cDNA samples derived from the un-induced PAO1::*p*LAC-*rep* strain using the absolute quantification method by qPCR. Calibrator standard curves for both genes were generated using genomic DNA extracted from the PAO1::*p*LAC-*rep* strain, whose concentration was determined using the QuantiFluor dsDNA System (Promega, USA). The copy number of *rep* and *rpoD* were calculated based on the gDNA calibrator standard curves and the mass of gDNA per genome.

### Molecular cloning & transposon mutagenesis

The *rep* gene with its presumed ribosomal binding site (5′-CGGAGG-3′) 5 bp upstream of the start codon (GTG) was PCR-amplified from pNUK73 plasmid DNA using the forward primer 5′-TAAGGATCCTGGCGGAGGCGGCT-3′ and reverse primer 5′- GCCAAGCTTTAACGTTCCCCTAACTT-3′. The PCR product was cloned downstream of *p*LAC between the BamHI and HindIII restriction sites in the multiple cloning site in the pUC18-mini-Tn7T-Gm-LAC (accession no. AY599234) delivery plasmid^24^. To integrate the IPTG-inducible *rep* construct into the mini-Tn7 site of the plasmid-free PAO1 parental strain used in this study, sucrose-treated PAO1 was cotransformed with the pUC18-mini-Tn7-*p*LAC-rep delivery vector and a Tn7 transposase-encoding helper plasmid pUX-BF13^48^ via electroporation to generate the PAO1::*p*LAC-*rep* strain. The negative control strain, PAO1::*p*LAC, which is isogenic to the PAO1::*p*LAC-*rep* strain except for the absence of *rep* downstream of *p*LAC, was created using the same procedure.

### GO term and pathway enrichment

GOEAST^49^ online tool (Customized-GOEAST, default parameter settings) was used to test for gene ontology enrichment among the group of differentially expressed genes. DAVID^50^ online tool was used to test for enrichment in several functional annotations.

### Similarity searches

To study horizontal gene transfer, we used several bioinformatic approaches that rely on similarity searches. First, we performed BLASTP similarity searches against the 48 Pseudomonas species genomes in the Pseudomonas Genome Database and all Pseudomonas species with complete genomes deposited in NCBI^51^. Second, we performed online BLASTP searches^52^ against the non-redundant database in NCBI. Finally, we performed online searches using the European Nucleotide Archive^53^, which accesses all Ensembl Genomes.

## Additional information

**Accession codes:** The sequences generated in this work have been deposited in the European Nucleotide Archive database under the accession code PRJEB8227.

**How to cite this article:** San Millan, A. *et al*. Interactions between horizontally acquired genes create a fitness cost in *Pseudomonas aeruginosa*. *Nat. Commun*. 6:6845 doi: 10.1038/ncomms7845 (2015).

## Supplementary Material

Supplementary Figures, Tables and References Supplementary Figures 1-4, Supplementary Table 1-3 and Supplementary References

Supplementary Data 1 Transcriptional profiles of the clones analysed by RNA-Seq. Relative expression of the different genes in PAO1 genome compared to the wild type PAO1 in: pNUK73 plasmid-carrying PAO1 (PAO1/pNUK73), and plasmid-carrying and plasmid-free PAO1 helicase mutant (PA1372) and kinase mutant (PA4673.15). Genes with significant changes in expression level (Wald Test Benjamini-Hochberg corrected p-value < 0.05, n= 5715) are shaded.

Supplementary Data 2 Functional enrichment analysis of differentially expressed genes in PAO1/pNUK73 compared to PAO1 using the DAVID and GOEAST software.
