# Supplementary material for: Interactions between horizontally acquired genes create a fitness cost in Pseudomonas aeruginosa
Source: Nat Commun. 2015 Apr 21;6:6845. doi: 10.1038/ncomms7845 (PMC4410645; doi:10.1038/ncomms7845)
Supplement: Supplementary Figures, Tables and References — Supplementary Figures 1-4, Supplementary Table 1-3 and Supplementary References [file ncomms7845-s1.pdf]

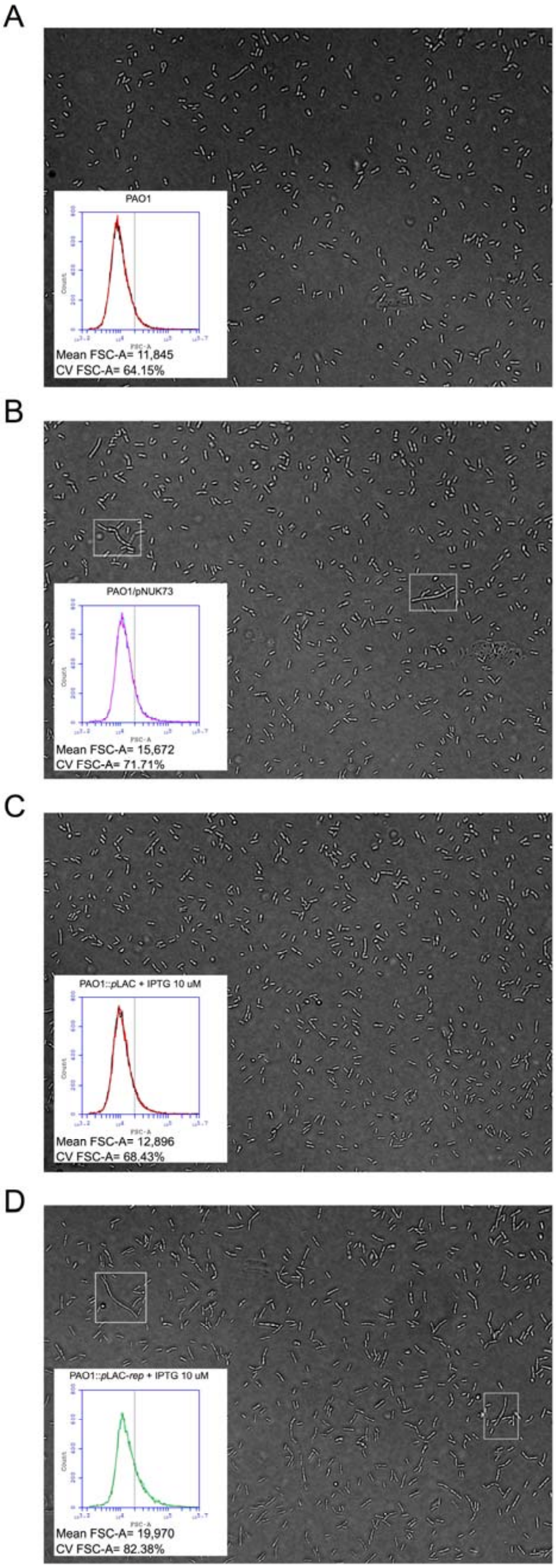

Supplementary Figure 1: Increase in cell size due to the expression of pNUK73 replication protein gene *rep*. The expression of the replication protein gene *rep* of pNUK73 produces an increase in cell size in PAO1. Each panel features an optical microscope image and a plot showing the distribution of Forward Scatter values (FSC-A) of three independent populations

of PAO1 (Panel A), PAO1/pNUK73 (Panel B), PAO1::*p*LAC + IPTG 10 µM (Panel C) and PAO1::*p*LAC-*rep* + IPTG 10 µM (Panel D). We measured 20,000 events for each population using flow cytometry and used FSC-A as a proxy for cell size. The mean FSC-A of PAO1 increases significantly in response to pNUK73 (Panels A and B; two-sample *t*-test: $P = 0.002$, $t = 8.78$, df= 3.3) and induced *rep* expression (Panels C and D; two-sample *t*-test: $P < 0.001$, $t = 48.7$, df= 3.6), which provides phenotypic evidence that the SOS response is activated in conjunction with *rep* expression. White boxes show elongated cells. The dashed line in the FSC-A plot is intended to highlight the higher asymmetry (skewness) in the right tail of the distribution as a result of a higher proportion of elongated cells in B and D compared to A and C, respectively.

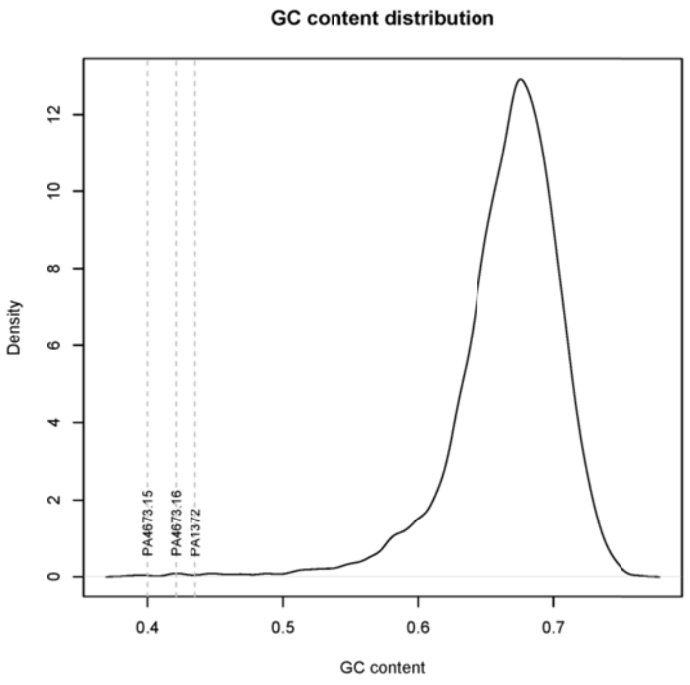

Supplementary Figure 2: Distribution of GC content in *P. aeruginosa* PAO1 genes. Chromosomal genes involved in compensatory adaptation to pNUK73 have very low GC content compared to PAO1 genome. Dotted lines indicate the GC content for the 3 genes carrying compensatory mutations responsible for the adaptation to the small plasmid pNUK73.

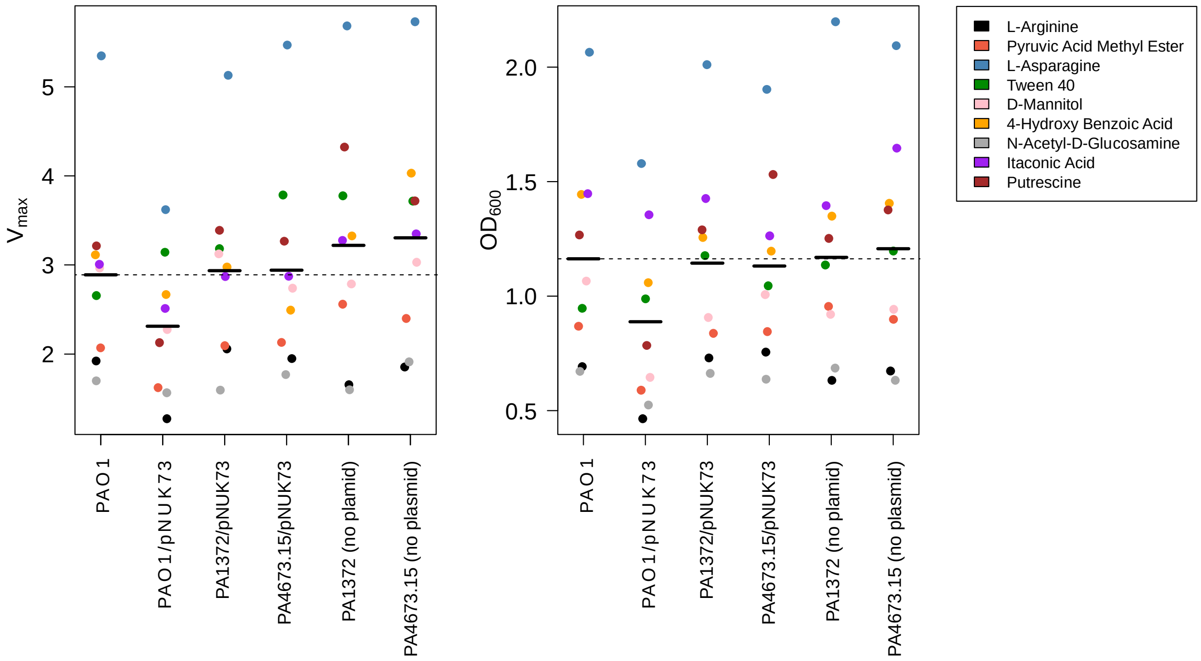

Supplementary Figure 3: Growth kinetics in Biolog EcoPlates. This graph shows the growth kinetics of PAO1, a helicase mutant clone (PA1372, premature stop codon at position 378) and a kinase mutant clone (PA4673.15, premature stop codon at position 95) with and without the plasmid pNUK73 in different carbon sources. We used the 31 different environments of the Biolog EcoPlates as the carbon sources. We observed measurable growth in 9 environments. The average of three independent measurements (five for the control PAO1) is represented by a dot in the chart. The black dashes represent the average value of V_{max} or OD_{max} for each clone and the dotted line represents the average for PAO1. Maximum velocity (V_{max}) and maximum OD_{600} (OD_{max}) of the clones in the different environments were compared to those of PAO1 as a proxy for bacterial fitness (growth rate and yield, respectively). PAO1/pNUK73 showed a decrease in both V_{max} and OD_{max} (Paired *t*-test, V_{max}: *P*= 0.021, *t*= 2.86; OD_{max}: *P*= 0.002, *t*= 4.49, df= 8) across the 9 environments. The helicase mutant (Paired *t*-test, V_{max}: *P*= 0.562, *t*= 0.60; OD_{max}: *P*= 0.667, *t*= 0.45, df= 8) and kinase mutant (Paired *t*-test, V_{max}: *P*= 0.752, *t*= 0.33; OD_{max}: *P*= 0.584, *t*= 0.57, df= 8) compensated for the cost of pNUK73 throughout the different environments. Finally, the plasmid-free helicase mutant showed no differences in V_{max} or OD_{max} (Paired *t*-test, V_{max}: *P*= 0.088, *t*= 1.94; OD_{max}: *P*= 0.841, *t*= 0.21, df= 8) compared to PAO1, while the plasmid-free kinase mutant presented no difference in OD_{max} and even a significant increase in V_{max} compared to PAO1 (Paired *t*-test, V_{max}: *P*= 0.009, *t*= 3.40; OD_{max}: *P*= 0.305, *t*= 1.10, df= 8). These results support the idea that the helicase and kinase have no clear role in *P. aeruginosa* PAO1.

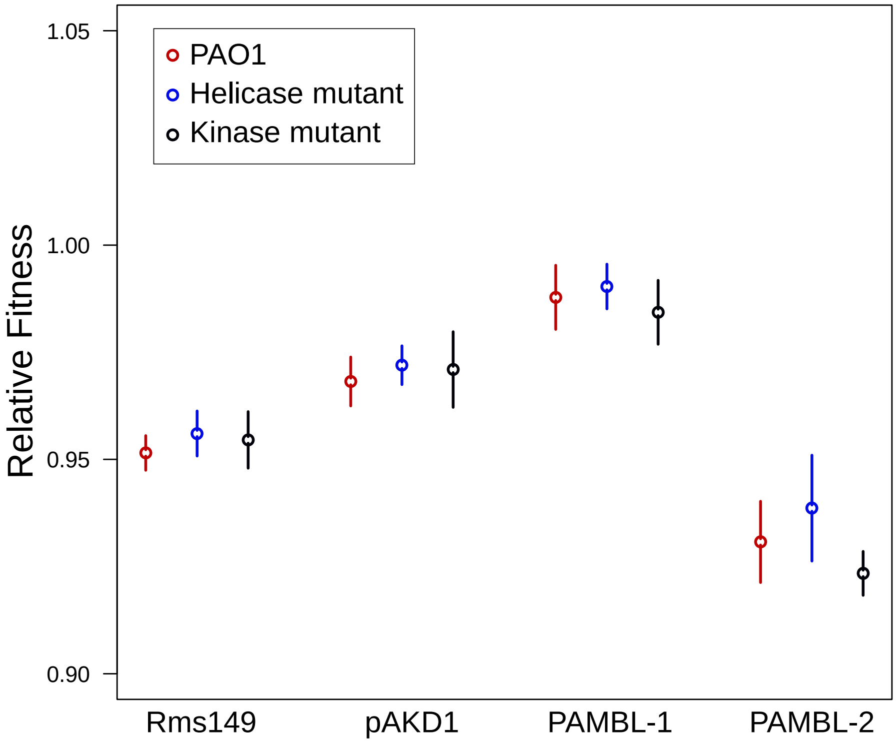

Supplementary Figure 4: Helicase and kinase mutations are not a general adaptation to plasmids. Compensatory mutations for pNUK73 do not compensate for the cost of all plasmids. Fitness (average ±SEM, N=3) relative to plasmid-free *P. aeruginosa* PAO1 of plasmid-carrying PAO1 (red), helicase mutant (PA1372, premature stop codon at position 378, blue), and kinase mutant (PA4673.15, premature stop codon at position 95, black). Four different plasmids were used: Rms149, pAKD1, PAMBL-1 and PAMBL-2. We performed nine biological replicates of the competition experiment for each clone. There were no significant differences in the fitness of the three different clones carrying a particular plasmid (ANOVA: Rms149, P= 0.69, F= 0.163, df= 1, 25; pAKD1, P= 0.762, F= 0.093, df= 1, 25; PAMBL-1, P= 0.711, F= 0.141, df= 1, 25; PAMBL-2, P= 0.5881, F= 0.302, df= 1, 25).

Supplementary Table 1: pNUK73 activates the SOS response and increased the relative expression levels of 15 genes of the SOS regulome (*p<0.05).

Gene locus in PAO1	Gene name	Fold-change in PAO1/pNUK73[1]	Benjamini-Hochberg corrected p-value
PA3008	*sulA*	5.3	1.25×10^{-6}*
PA3007	*lexA*	4.9	1.37×10^{-6}*
PA3413	*yebG*	3.7	8.07×10^{-4}*
PA4763	*recN*	4.5	3.88×10^{-6}*
PA2288		7.7	3.93×10^{-9}*
PA3414		6.5	4.72×10^{-7}*
PA3617	*recA*	3.8	8.25×10^{-5}*
PA3616	*recX*	5.6	1.09×10^{-6}*
PA1044		3.4	0.0165*
PA1045	*dinG*	2.2	0.0541
PA0069	*phl*	1.6	0.4800
PA0922		5.8	3.02×10^{-7}*
PA0669	*dnaE2*	1.3	0.6731
PA0670	*imuB*	1.5	0.5502
PA0671	*sulA2*	1.3	0.7091

[1] Relative to the plasmid-free PAO1 wild-type strain.

Supplementary Table 2: Bacterial strains used for transcriptome profiling by RNA-Seq and quantitative real-time PCR.

Strain name	Descriptions	Biological replicate	RNA integrity (RIN)
PAO1	*P. aeruginosa* PAO1 wild-type strain	1	8.9
		2	9.9
PAO1/pNUK73	pNUK73 plasmid-bearing PAO1 strain	1	8.9
		2	9.7
PA1372 helicase mutant /pNUK73	PAO1 strain with a mutation compensating for the cost of PAO1 (PA1372, premature stop codon at position 378) carrying plasmid pNUK73	1	8.9
		2	9.9
PA1372 helicase mutant	PAO1 strain with a mutation compensating for the cost of PAO1 (PA1372, premature stop codon at position 378) without plasmid pNUK73	1	9.0
		2	9.9
PA4673.15 kinase mutant /pNUK73	PAO1 strain with a mutation compensating for the cost of PAO1 (PA4673.15, premature stop codon at position 95) carrying plasmid pNUK73	1	9.8
		2	9.9
PA4673.15 kinase mutant	PAO1 strain with a mutation compensating for the cost of PAO1 (PA4673.15, premature stop codon at position 95) without plasmid pNUK73	1	9.9
		2	9.9
PAO1::*p*LAC-*rep*	PAO1 strain with an IPTG-inducible *rep* gene downstream of the *lac* operon promoter inserted into the mini-Tn7 chromosomal site.	Not applicable	
PAO1::*p*LAC	Negative control strain that is isogenic to PAO1::mini-Tn7-Gm-*LAC*-*rep*, but lacks *rep* downstream of the *lac* operon promoter.	Not applicable	

Supplementary Table 3: Oligonucleotide sequences and amplification efficiency of quantitative real-time PCR primers used in this study

Gene	Forward primer (5' → 3')	Reverse primer (5' → 3')	Amplification efficiency (%)	Reference
acpP	ACTCGGCGTGAAGGAAGAAG	CGACGGTGTCAAGGGAGT	84.3	[1]
atpA	TACGCCAGATCACGCCCGGA	GGCGGCGTCTACGGTATGGC	83.0	[2]
rpoD	CAGGTGGCGTAGGTGGAGAA	GGGCGAAGAAGGAAATGGTC	90.5	[3]
lexA	ATCGGAGCGAACTCAGGGT	CCGTATCAATCCCGCCTTCT	82.2	This study
recA	TTCGCTACCCACCACCTCGT	CAAGAACGCCAACTGCCTGG	81.1	
rep	AGCAGAGCGACGAGGAAGTG	ATAGCCACCCAGCCGCCATT	84.5	

The coefficients of correlation (R^2) for all primer pairs are in the range of $0.995 < R^2 < 1$. The dissociation curve of each final qPCR product showed a single peak, confirming the specificity of each reaction.

Supplementary References

1. Lenz AP, Williamson KS, Pitts B, Stewart PS, Franklin MJ. Localized gene expression in Pseudomonas aeruginosa biofilms. *Appl Environ Microbiol* **74**, 4463-4471 (2008).

2. Qi Q, Preston GM, MacLean RC. Linking system-wide impacts of RNA polymerase mutations to the fitness cost of rifampin resistance in Pseudomonas aeruginosa. *MBio* **5**, e01562 (2014).

3. Savli H, Karadenizli A, Kolayli F, Gundes S, Ozbek U, Vahaboglu H. Expression stability of six housekeeping genes: A proposal for resistance gene quantification studies of Pseudomonas aeruginosa by real-time quantitative RT-PCR. *J Med Microbiol* **52**, 403-408 (2003).
